# Occupational Silica Exposure and Dose–Response for Related Disorders—Silicosis, Pulmonary TB, AIDs and Renal Diseases: Results of a 15-Year Israeli Surveillance

**DOI:** 10.3390/ijerph192215010

**Published:** 2022-11-15

**Authors:** Rachel Raanan, Oren Zack, Maya Ruben, Idan Perluk, Shlomo Moshe

**Affiliations:** 1 The Department of Environmental and Occupational Health, School of Public Health, Sackler Faculty of Medicine, Tel-Aviv University, Tel-Aviv 6997801, Israel; 2 Public Health Services, Ministry of Health, Jerusalem 9446724, Israel; 3 The Department of Occupational Medicine, Hashfela and Jerusalem District, Maccabi Healthcare Services, Rishon Letzion 7505001, Israel

**Keywords:** autoimmune disorders (AIDs), crystalline silica exposure, renal disease, silicosis

## Abstract

Background: The exposure patterns of respirable crystalline silica based on environmental records, as well as the link to different diseases, are not well described. Aims and objectives: In this study, we evaluated the risk for various diseases in relation to occupational silica exposure, including Silicosis, pulmonary tuberculosis (TB), Autoimmune disorders (AIDs) and Renal diseases. Methods: We assessed the relationship between silica exposure and the rate of various diseases such as silicosis, pulmonary TB, AIDs and renal diseases in a cross-sectional study. We reviewed the medical records and exposure level of workers exposed to silica during the past two decades. Results: 261 workers were included in the study, total duration of exposure 15.6 years (±SD 8.74); 42.15% of them were employed in the artificial marble industry and 29.5% in manufacturing and construction industries. The average yearly silica exposure levels were 0.23 mg/m^3^ (±0.34). The average cumulative silica concentration was 3.59 mg/m^3^/y (±4.80). We found 25 (9.58%) incident cases of silicosis, 10 cases of chronic obstructive pulmonary disease (COPD) and emphysema (3.83%), six cases of several AIDs (2.30%), five cases of pulmonary TB (1.92%), three cases of renal diseases (1.15%), two cases of sarcoidosis (0.77%) and no lung cancer cases. When compared to studies with the same endpoint we found excess risk of silicosis (RR = 2.67/0.13 = 20.5, 95% CI 9.85 to 42.86)), pulmonary TB (RR = 30.70, CI 3.43–274.49, *p* = 0.002) and AIDs (RR = 2.87, 95% CI = 1.27 to 6.48 *p* = 0.01). Conclusions: Silica exposure was a significant risk factor for silicosis, pulmonary TB and AIDs. Our findings are important given persistent worldwide silica-related epidemics in low and high-income countries.

## 1. Introduction

Silica is considered to be the most abundant mineral on earth. Nowadays, there are an estimated 23 million workers in China, 11.5 million in India, 3.2 million in the European Union and 2.3 million in the United States (US) who are occupationally exposed to respirable crystalline silica (RCS) or quartz [1].

Silicosis is a fibrotic respiratory disease caused by the inhalation and deposition of RCS. The use of proper protective measures can reduce its incidence and even prevent it altogether by significantly reducing the concentration of inhaled dust [1,2,3]. The worldwide incidence of silicosis is increasing due to the high demand for occupations that involve silica exposure and the lack of proper protection [1,2,3,4]. In recent years, outbreaks of silicosis have also been reported in developed countries in various occupations. The increasing use of artificial marble, composed of 85–93% RCS, is causing a notable rise in silica exposure, which in turn leads to a major increase in the number of silicosis cases, reported mainly in Spain, Australia, and Israel [5].

The threshold limit value (TLV) for RCS in Israel is 0.1 mg/m^3^. According to the Israeli regulations, silica workers are required to undergo biennial medical surveillance that includes a medical examination, pulmonary function tests and a chest X-ray every five years. Medical surveillance data is recorded in the patient records within their health maintenance organizations (HMOs).

Besides silicosis, silica exposure may cause various other diseases. A correlation between increased silica exposure and development of pulmonary tuberculosis (TB) has previously been reported [6,7,8,9,10]. Chronic obstructive pulmonary disease (COPD) has also been associated with silica exposure, independent of smoking [8]. The International Agency for Research on Cancer (IARC) classified free silica as a group 1 carcinogen that induces lung cancer in cases of background silicosis [11,12]. Some articles indicated a possible association between silica and several autoimmune disorders (AIDs). Most of these studies focused on the relationship between silica exposure and rheumatoid arthritis (RA) [8,13,14]. Another group of medical conditions suggested to be associated with silica exposure are renal disorders [15,16,17].

Although there is some evidence of an association between silica exposure and the incidence of various disorders [10,18], the absence of reliable silica quantitative dosimetric data, the relative rarity of these outcomes and the fact that most evidence reported has been statistically non-significant, calls for further research, which is especially crucial given the persistent worldwide silica-related epidemics in both low and high-income countries [17]. In this study, we examined the association between silica exposure levels and various potential outcomes.

## 2. Methods

### 2.1. Study Setting, Participants, and Design

**Figure 1:**
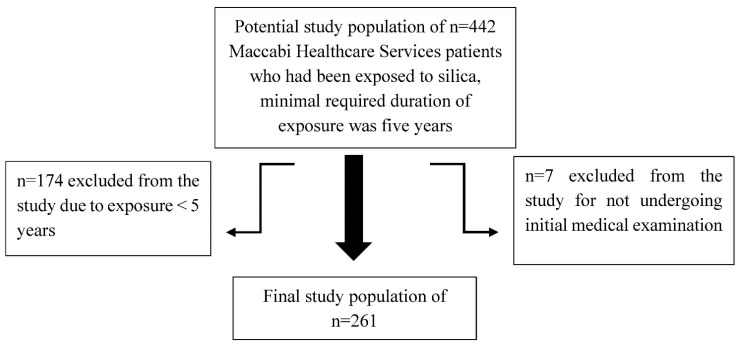
Flowchart of the study selection process.

We collected data on all patients covered under Maccabi Healthcare Services (MHS) who had been occupationally exposed to silica. Data were retrieved from medical records through the first half of 2019. Average year of first exposure was 2003 (SD = 8.54, range 1972 to 2014). The inclusion criteria required minimum duration of exposure of at least five years. Patients who had not undergone an initial periodical medical examination when they first started working in an exposed area were excluded. A total of 442 patient records were collected, 261 of which met the study criteria, as described in Figure 1. Data was retrieved by an OP practitioner and analyzed via a unique assigned serial number (not an ID number). Thus, personal data are kept only in MHS. The research protocols were approved by the MHS Review Board, approval number 0044/18-BBL.

### 2.2. Medical Records and Morbidity Assessment

From each medical record, we extracted demographic data, socio-demographic data, and patient’s medical history. Occupational information included industry details, duration of employment, type of work, associated exposures, and use of personal protective equipment (PPE). Medical information included height and weight, respiratory symptoms, smoking history (yes/no) and amount of smoking (packs/years), relevant family history and use of medications–namely respiratory and/or inhaled steroids. Relevant morbidities noted were silicosis, COPD, AIDs, lung cancer, kidney diseases and pulmonary TB.

### 2.3. Environmental Monitoring Measurements

We collected environmental monitoring data for each of the applicable plants and departments to estimate individual exposure levels. We recorded all available measurements of RCS (mg/m^3^). We also documented the worker’s specific occupation, the first year of employment for all occupations involving RCS and the time since work initiation (years). In order to be able to compare between different studies we used two parameters, Cumulative RCS Exposure (CRCSE) and CRCSE Silicosis Rate—Cumulative Silicosis Rate (CSR). The term CRCSE is defined as the average RCS in mg/m^3^ multiplied by the exposure period (years) and measured in mg/m^3^/y. The CSR is the silicosis rate in a study/CRCSE and measured in % silicosis rate/mg/m^3^/y.

### 2.4. Data Analysis

For each participant, we calculated annual exposure for each available year, RCS weighted average annual exposure and CRCSE. For 67 of the study subjects, we found no records of environmental monitoring throughout the years of their employment. In order to assess the exposure as accurately as possible, the measurement value was adjusted according to the worker’s specific work station; otherwise, we made an average of the plant’s three highest measurements, with maximum adjustment to the worker’s occupation and work station. In order to estimate the weighted average annual exposure, we calculated the aggregate of the yearly measurements divided by the number of exposure years. If a participant did not have a recorded measurement for every year of work, the first recorded value was used for the preceding years until the next available recorded measurement.

We analyzed our data using bivariate analyses to describe general demographic data, the scope of exposure, duration of employment and smoking. We then examined the incidence of comorbidities and compared it to the incidence described in the literature and calculated the relative risk (RR) compared to various populations. We analyzed the data using Stata (version IC13.0; StataCorp, College Station, TX, USA). We set statistical significance at *p* < 0.05 for all analyses.

## 3. Results

Characteristics of study participants are shown in Table 1. The final study population (see Figure 1 for exclusion criteria) included 261 workers. Most study participants were men (*n* = 252, 96.6%). The participants were primarily Jewish (81.6%) and 15.3% were Arabic. Most participants were current (42.9%) or past (12.7%) smokers. The average smoking exposure in pack years was 18.8 (SD = 13.4, range 2 to 64). Only one participant reported asbestos exposure (0.4%).

The employment characteristics of participants are shown in Table 2. Average age at first exposure was 36.3 years (SD = ±11.0; range 14 to 67) and average year of first exposure was 2003 (SD = ±8.54, range 1972 to 2014). Duration of workers’ exposure to silica ranged between 5 to 45 years (average = 15.6; SD = ±8.74). The average yearly RCS exposure levels were 0.23 mg/m^3^ (SD = ±0.34; range 0.02 to 1.97), above the Israeli TLV (0.1 mg/m^3^). The CRCSE was 3.59 mg/m^3^/y (SD = ±4.80). Most study participants were employed in either the artificial marble industry (42.15%) or construction materials manufacturing (29.5%).

Morbidity and total silica exposure data for each disease of interest are shown in Table 3. We found 25 (9.58%) incident cases of silicosis, 10 cases of COPD and emphysema (3.83%) 9/10 current or ex-smokers (90%, not shown), five cases of pulmonary TB (1.92%), six cases of several AIDs (2.30%), three cases of renal disease (1.15%), two cases of sarcoidosis (0.77%) and no lung cancer cases (Table 3). The duration of exposure until diagnosis of silicosis (years) was higher than in non-silicosis workers (16.04 ± 7.13 vs. 15.13 ± 8.6, respectively *p* < 0.05), average RCS was not significantly different among the groups (0.42 ± 0.52 vs. 0.22 ± 0.32, respectively *p* = 0.075), and CRCSE was higher in silicosis workers’ group (7.60 ± 10.69 versus 2.66 ± 3.58, respectively *p* < 0.05).

Associations between the study population and rate of various diseases of interest compared to the general population are presented in Table 4. Comparison to the unexposed population revealed that the relative risk was higher for pulmonary TB (RR = 30.70, 95% CI 3.43–274.49, *p* = 0.002) and for AIDs (RR = 2.87, 95% CI = 1.27–6.48 *p* = 0.01). The silicosis rate was higher than most studies documented in the literature (see Table 5).

## 4. Discussion

This cross-sectional study included 261 subjects, followed up for 15.6 years with an average yearly exposure of RCS 0.23 ± 0.34 mg/m^3^/y. The CRCSE was 3.59 ± 4.80 mg/m^3^/y. The study sample included 25 (9.58%) incident cases of silicosis, 10 cases of COPD and emphysema (3.83%), six cases of several AIDs (2.30%), five cases of pulmonary TB (1.92%), three cases of renal disease (1.15%), two cases of sarcoidosis (0.77%) and no lung cancer cases. The silicosis morbidity rate was as expected in other cohorts (silicosis rate of 2.67/1 mg^3^/years). The RR for pulmonary TB and AIDs was high and statistically significant as well.

Our study has several strengths. The medical data were retrieved from the MHS medical registry. These data are complete and enabled us to locate all medical diagnoses including cases where the worker has not been employed in a silica-exposed industry. In addition, exposure data have been collected from environmental monitoring of all working places; i.e., as described in the Section 2, exposure data for most participants in our study are accurate.

This study has several limitations and biases. Our sample includes only 261 workers. The subjects selected for the study were only those whose had periodical health examination follow up in MHC. There may be differences between the HMOs in the quality of follow-up of silica-exposed patients, in the frequency of medical checkups and tests, and consequently, in the accuracy of diagnosis. Additionally, a healthy worker effect bias may have occurred. Our study sample is comprised of people employed in manual labor, which demands higher-than-average physical abilities; thus, it may exclude subjects with existing or acquired background morbidities. Only workers who had periodic health examinations were included in our study, so they were not biased by whether they had a disease or not. The disease was found in the complete medical registry of MHC which we had. The recall bias (to disease or exposure) was refrained by using complete medical database and exposure database of the Israeli Health of Safety administration.

### 4.1. Silicosis

Many factors influence the extent of silicosis in exposed groups, including CRCSE (a function of intensity and duration often expressed as mg/m^3^/y), length of follow-up from first exposure and potency factors, type and use of PPE [23], type of exposure [18,23], and type of industry [7]. A review article tried to assess the risk of developing silicosis after 45 years of exposure to RCS at a concentration of 0.1 mg/m^3^/y. The reviewed studies varied in follow-up time and the studied industry, and their findings ranged between risks of 1% and 80% [13]. Such a high variation in findings suggests that the risk of developing silicosis does not increase linearly as the exposure increases, but rises more steeply at very high exposure levels [22]. To compare results provided from different studies we used the CRCSE and the CSR parameter, the latter being 2.67% cases/mg/m^3^/y (Table 5).

Churchyard et al. [19] studied South African gold miners with an average RCS exposure level of 0.053 mg/m^3^/y, long duration of exposure (21.8 years), and 19% prevalence of silicosis. The total crude proportions with silicosis at autopsy of miners were 8% among people of Color and 14% among Caucasians.

Chen et al. [18] analyzed data of male cohorts and included 4028 tin miners (group 1), 14,427 tungsten miners (group 2), and 4547 pottery workers (group 3), who had similar onset of employment and duration of follow-up. The average intensity of RCS was 8.2, 3.9, and 4.0 mg^3^/year in groups 1,2,3, respectively, and the cumulative total dust exposure (205.6, 62.3, 64.6 mg/m^3^/y in groups 1,2,3, respectively), which was very high in all three groups. However, quite surprisingly, the CSR was very low as compared to other studies (0.08, 0.34, 0.3% cases/mg/m^3^/year in groups 1,2,3, respectively). Steenland [20] summarized data from studies with adequate follow-up, and concluded that a lifetime 45-year exposure at the standard of 0.1 mg/m^3^ of RCS will result in a risk of silicosis (defined as an X-ray with ILO category 1/1 or greater) of 47% to 77% (average—62%). In these studies, the CRCSE was 4.5%/mg/m^3^/y and the CSR was 13.78% cases/mg/m^3^/y.

In the UK between 1996 and 2017, about 500,000 workers were exposed to silica, and 216 cases of silicosis have been reported to a surveillance program and an estimated 700 cases were reported for the country as a whole. The average follow-up period was 22 years. The prevalence of 0.14% seems very low as compared to other countries’ estimations [21].

In an overview in Table 5 it can be observed that it is reasonable to find higher estimates of silicosis when the endpoints of diagnosis in the study are an autopsy [22] or an X-ray diagnosis [19,20,23]. This ultimately increases the number of diagnosed workers, since in many cases CS is asymptomatic [7]. The CRCSE in our study is somewhere in between the reviewed studies. Compared to the clinical report by Barber et al. [21], the CSR in our study is much higher (RR = 2.67/0.03 = 20.5 ± SD). Barnes et al. [5] reviewed all published case reports and longitudinal cohort studies of silicosis in modern workplaces, 33 in total, with eight of them concerning artificial stone benchtop manufacturing. The average age was 33–47 years (50 in the current study) and average duration of exposure was 10–20 years (16 in the current study). In high-risk occupations (like the marble industry), the reported incidence of silicosis is upwards of 50–60% and the mortality rate is 10–100%, both exceedingly higher than the prevalence and mortality rates of CS than in more traditional occupations such as the mining industry (mortality of 6 per 1000 workers). We are aware that most workers in Israel are immigrants from Russia, and this fact, along with other parameters like under-reporting, high exposure, differences in industry and differences in the use of PPE could explain the differences.

If we assume that a worker is exposed to 0.1 mg/m^3^/y (TLV in many countries) we can predict that after 10 years (i.e., CRCSE =1 mg/m^3^/y), according to our data, 2.67% of workers will develop silicosis and that the prevalence will be doubled after 20 years. This implies that a TLV of 0.1 mg/m^3^ is not protective enough and should be decreased to 0.05 mg/m^3^.

### 4.2. Pulmonary Tuberculosis and Silica Exposure

In our study, we found 5/261 (1.92%) workers diagnosed with pulmonary TB during an average follow-up period of 15.6 years. Accordingly, the annual pulmonary TB incidence rate is 0.12% or 12.28/100,000. The Israeli national average incidence rate of pulmonary TB between 2008–2018 was 3–4/100,000 [24]. According to these data, the risk of pulmonary TB in our sample compared to the general population was 30.70 (CI 3.43–274.49, *p* = 0.002).

The contribution of silica exposure to the risk of pulmonary TB has been examined in multiple studies and varies according to the studied population and the general prevalence of pulmonary TB in the studied area. For example, in the US, the estimated burden for pulmonary TB among the silica-exposed workers ranges from 3.2% to 4.9%, while in a South African population of miners the burden is estimated at ~2.3% [6]. Another study, which reviewed death certificates from 27 US states, showed that the odds ratio of developing pulmonary TB increased along with silica exposure; for example, for exposure levels defined as low, the odds ratio was 1.47, but it rose to 1.6 and 2.48 at high and very high exposures, respectively [8].

Based on the WHO pulmonary TB fact sheet [25], the estimated worldwide rate of pulmonary TB in the general population is about 0.13%. In contrast, literature shows that in silica-exposed populations this rate is three times higher and is estimated at approximately 0.39%. The incidence of pulmonary TB in Israel is rather low and is estimated at approximately 3–4/100,000 people [24]. However, in our sample we found five cases of pulmonary TB—approximately 1.9%; this is a very high rate compared to the general population both in Israel and worldwide. We are aware that workers employed in the marble industry are usually Russian Jews, uneducated, smokers and of low sociodemographic status. These parameters combined with silica exposure can explain the very high rate of pulmonary TB in this group. Our findings support the association described in the literature between silica exposure and pulmonary TB.

### 4.3. Autoimmune Disorders

Our study assessed incidence rates of various AIDs among a sample of silica-exposed workers. Mounting evidence indicates that silica exposure can cause AIDs, particularly rheumatoid arthritis (RA), systemic lupus erythematosis (SLE) and scleroderma. Autoimmune morbidity is determined by multiple genetic and environmental factors. Therefore, establishing an association between AIDs and silica exposure is challenging. Furthermore, rates of AIDs are higher among women, while the majority of silica-exposed individuals are men. Thus, the rate of such diseases among occupational samples is low, and achieving statistical significance is challenging [13,14]. Additionally, the prevalence of most AIDs is low [26,27]. Thus, it may be best to examine the total amount of all AIDs in a relatively big sample size. For example, Eaton et al. [28] investigated the whole population of Denmark and demonstrated a sizable prevalence of AIDs of approximately 5%. Cooper et al. [26] concluded that the overall prevalence of AIDs is at least 3% in the US population and that most AIDs disproportionately affect women, with approximately 80% of patients being female.

The association of silicosis exposure with RA has been examined in various studies, some of which have found an association even though most ignored relevant confounders such as smoking and family history. Boudigaard et al. [14] found that men exposed to high levels of RCS compared with non-exposed men presented with an increased incidence rate ratio calculated for four AIDs (IRR = 1.53, 1.62, and 1.57 for total AIDs, systemic sclerosis, and RA, respectively). The overall risk increased with cumulative exposure attained since entering the workforce (IRR = 1.07 95% CI 1.05–1.09 per 50 mg/m^3^/y). Khuder et al. [27] reviewed 15 studies published from 1986 to 2001 in a meta-analysis and found a combined RR for RA and silica exposure of 3.43, (95% CI 2.25–5.22). Cooper et al. [29] also provide an overview of the literature, citing five occupational cohort studies with a RR of ≥3.

Our sample, which consists of 96.6% males included six diagnosed cases of AIDs (2.3% of the sample). Under the assumption that the prevalence of AIDs is ~4% and that ~80% of AIDs cases are female, we conclude that the expected prevalence in our sample is 0.80% and the RR is 2.87 (95% CI = 1.27 to 6.48), similar to previous studies [14,28,29]. Thus, like other studies in this field, our study indicates an association between silica exposure and AIDs.

## 5. Conclusions

In this cross-sectional study, we evaluated the risk of silica exposure in an average follow-up of 15.6 years with an average yearly exposure of 0.23 ± 0.34 mg/m^3^/y RCS. When compared to studies with the same endpoint we found an excess risk of silicosis (RR = 2.67/0.13 = 20.54, 95% CI 9.85–42.86, *p* < 0.001). A comparison to the unexposed population revealed that the excess risk was higher for pulmonary TB (RR = 30.70, CI 3.43–274.49, *p* = 0.002) and AIDs (RR = 2.87, CI= 1.27–6.48 *p* = 0.01). We also found that exposure to 0.1 mg/m^3^/y can predict that after 10 years, 2.67% of workers will develop silicosis and that the incidence will be doubled after 20 years. This implies that the TLV of 0.1 mg/m^3^ is not protective enough and should be decreased to 0.05 mg/m^3^. This study demonstrates the importance of primary prevention techniques such as use of PPE, environmental ventilation and monitoring and use of wet techniques in combination with secondary prevention like health surveillance programs and fitness for work with limitations in order to lower workers’ exposure from 0.23 mg/m^3^ to 0.05 mg/m^3^ [24,30,31]. More research is needed to prevent silicosis and other diseases and better protect those workers.

## Figures and Tables

**Table 1:** Participants’ demographic and clinical characteristics *.

Characteristics	N (%), Mean [SD], Range, Years
Age, mean [SD], range, years	50.1 (N = 253) [11.1], 23 to 76
Sex, No., %	
Male	252 (96.55)
Female	9 (3.45)
Nationality, No., %	
Jewish	213 (81.61)
Arabic	40 (15.33)
Other	8 (3.07)
Smoking **	
Yes	108 (42.86)
No	112 (44.44)
Past smoker	32 (12.70)
Duration mean [SD], range, pack years	18.8 (N = 116) [13.4], 2 to 64
Asbestos exposure, No., %	1 (0.38)
Total, No., %	261 (100)

* Data is presented as either mean (SD) or frequency (%). ** Unknown, *n* = 9.

**Table 2:** Employment characteristics of the participants.

Characteristics	Mean [SD], Range, Years	N (%)
Year of initial exposure, mean [SD], range, years	2003.18 [8.54], 1972 to 2014	260
Age at initial exposure, mean [SD], range, years	36.3 [11.0], 14 to 67	253
Duration of exposure, mean [SD], range, years	15.6 [8.74], 5 to 45	260
Average intensity respirable quartz (mg/m^3^/y)	0.23 [0.34], 0.02 to 1.97	219
CRCSE * (mg/m^3^/y)	3.59 [4.80], 0.11–37.43	219
Type of Industry		
Artificial marble, No., %		110 (42.15)
Construction material manufacturing, No., %		77 (29.50)
Steel, No., %		21 (8.05)
Mining, No., %		15 (5.75)
Coal, No., %		14 (5.36)
Construction, No., %		7 (2.68)
Sand blasting, No., %		1 (0.38)
Other **, No., %		6 (2.30)
Unknown		10 (3.83)

* CRCSE—Cumulative Respiratory Crystalline Silica exposure. ** *n* = 2 dental laboratories, *n* = 1 plastic, *n* = 1 plasterwork, *n* = 2 ceramics.

**Table 3:** Morbidity and total silica exposure for each disease of interest.

Morbidity, Mean [SD], Range (*n*)	N (%)		Average RCS * mg/m^3^/y), Mean [SD], Range, *n*	CRCSE ** (mg/m^3^/y), Mean [SD], Range, *n*
Silicosis	25 (9.58)	16.04 [7.13], 4 to 31 (23)	0.42 [0.52],0.02 to 2.01 (18)	7.60 [10.69], 0.34 to 37.43 (20)
Pulmonary Tuberculosis	5 (1.92)	17.25 [5.56], 11 to 24 (4)	0.05 (*n* = 1)	1.15 [0.06], 1.06 to 1.19 (2)
Autoimmune disorders	6 (2.30)	12.0 [7.55], 5 to 20 (3)	0.11 [0.09], 0.02 to 0.2 (3)	2.21 [2.69], 0.49 to 6.85 (5)
Renal	3 (1.15)	11.0 [7.55], 4 to 19 (3)	0.57 [0.73], 0.03 to 1.40 (3)	3.72 [2.96], 0.31 to 5.59 (3)
COPD and emphysema	10 (3.83)	11.0 [5.83], 6 to 21 (6)	0.53 [0.51], 0.05 to 1.21 (4)	2.96 [2.66], 0.75 to 7.28 (8)
Sarcoidosis	2 (0.77)	21.5 [0.71], 21 to 22 (2)	0.23 (---) *n* = 1	5.14 (---) *n* = 1
Non-silicosis workers	236 (90.42)	15.13 [8.6]	0.22 [0.32]	2.66 [3.58]

* RCS—Respirable Crystalline Silica; ** CRCSE—Cumulative Respiratory Crystalline Silica Exposure.

**Table 4:** Associations between silica exposure and rate of various diseases of interest compared to the general population.

Morbidity	N (%)	Mean Age at Diagnosis (Years) [±SD]	% of Smokers	Mean PYS * [±SD]	Expected Rates of Disease (%) *	RR [95% CI], *p* Value
Pulmonary TB	5 (1.92)	48.25 [7.46]	80	22 [14.99]	0.004 (ISR)	30.70 [3.43–274.49], *p* = 0.002
Autoimmune disorders	6 (2.30)	50.67 [8.50]	66.67	22.33 [6.81]	0.8 (WW)	2.87 [1.27–6.48], *p* = 0.01
Renal	3 (1.15)	46.33 [4.04]	66.67 **	34	2 (WW)	--
COPD and emphysema	10 (3.83)	46.25 [12.49]	90 ****	28.57 [22.53]	--	--
Sarcoidosis	2 (0.77)	50.5	50	15	--	
Lung cancer	0	--	--	--	7.5 (WW)	--

PYS—pack years of smoking. * Worldwide (WW); Israel (ISR). ** 33.33 current smokers; 33.33 past smokers. **** 50% current smokers; 40% past smokers.

**Table 5:** Silicosis rate in different industries according to CRCSE.

Author (Ref.)	Year of Publication	Country	Type of Industry	Exposed Workers (*n*)	Exposure Data	Duration of Exposure (Years)	CRCSE	% Cases of Silicosis	Method of Diagnosis	CSR
Rosenman et al. [15]	2003	USA	Mining	121,000 *	0.29	7.40	2.16	6.0	Death certificates	2.78
Chen et al. [18]	2005	China	Pottery workers	4547	8.20	24.90	205.60	17.3	X-ray	0.08
Chen et al. [18]	2005	China	Tin miners	4028	3.90	16.40	62.30	21.2	X-ray	0.34
Chen et al. [18]	2005	China	Tungsten miners	14,427	4.00	16.50	64.60	19.5	X-ray	0.30
Churchyard et al. [19]	2004	South Africa	Mining	520	0.05	21.00	1.11	19.0	X-ray	17.07
Steenland et al. [20]	2005	USA	Miners		0.10	45.00	4.50	62.0	X-ray	13.78
Barber et al. [21]	2019	UK	Various industries	500,000 *	0.05	22.00	1.10	0.1	Physicians’ report	0.13
Nelson et al. [22]	2010	South Africa	Mining—Coloured	18,276 *	1.00	7.70	7.70	21.0	Autopsy	2.73
Nelson et al. [22]	2010	South Africa	Mining—Caucasians	19,842 *	0.05	17.10	0.86	27.0	Autopsy	31.58
Raanan et al. [Current Study]	2022	Israel	Various industries	261	0.23	15.60	3.59	9.6	Clinical cases	2.67

* Estimated exposure—Using data retrieved from IARC monograph 100c [12]. Exposure data—Average intensity respirable quartz (mg/mg^3^/y). CRCSE—Cumulative Respiratory Crystalline Silica Exposure is calculated for the full duration of exposure (mg/m^3^/y). CSR—Cumulative Silicosis Rate (% of cases per mg/m^3^/y).

## Data Availability

The data underlying this article are available in the article.

## Abbreviations

AIDs: Autoimmune disorders; CI: Confidence interval; COPD: Chronic obstructive pulmonary disease; CRCSE: Cumulative RCS exposure CSR: Cumulative silicosis rate; HMOs: Health maintenance organizations; IARC: International Agency for Research on Cancer; MHS: Maccabi Healthcare Services; OR: Odds Ratio; RCS: Respirable crystalline silica; RA: Rheumatoid arthritis; RR: Relative risk; SLE: Systemic Lupus Erythematosis; TB: Tuberculosis; TLV: Threshold limit value.
